# Exploring Splicing‐Energy Axis Associations to Diet and Longevity

**DOI:** 10.1111/acel.70335

**Published:** 2025-12-28

**Authors:** Stefano Donega, Myriam Gorospe, Luigi Ferrucci

**Affiliations:** ^1^ Translational Gerontology Branch National Institute on Aging, NIH Baltimore Maryland USA; ^2^ Laboratory of Genetics and Genomics National Institute on Aging, NIH Baltimore Maryland USA

## Abstract

There is increasing evidence that nutrient composition, even without lowering total calorie intake, can shape lifespan through mechanisms independent of mitochondrial regulation. Brandon and colleagues recently reported that a low‐protein, high‐carbohydrate (LPHC) diet enriched with non‐digestible cellulose, extends lifespan in mice by shifting the liver proteome through altered RNA splicing, a response different from the mitochondrial improvements typically seen with caloric restriction. The authors' findings support the “energy‐splicing resilience axis,” which proposes that changes in splicing help cells adapt to energetic and nutritional stress. We discuss how diet influences spliceosomal components such as SRSF1, linking nutrient sensing, AMPK signaling, and tissue‐specific resilience pathways. We also consider the splicing paradox in aging, where beneficial isoforms increase despite a concomitant increase in splicing errors. Understanding how dietary and pharmacologic interventions modulate splicing may shed light on strategies to maintain homeostatic proteomes and support healthy longevity.

## Graphical abstract

Caloric restriction improves mitochondrial function, while a low‐protein, high‐carbohydrate diet improves pre‐RNA splicing, as shown by Brandon et al. Despite employing distinct pathways, both dietary strategies enhance the energy‐splicing axis to increase longevity.

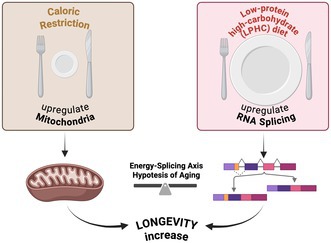

In a recent article published in Aging Cell (Brandon et al. 2025), Brandon and colleagues report that a low‐protein, high‐carbohydrate (LPHC) diet supplemented with non‐digestible cellulose extends lifespan in mice with a similar effect to caloric restriction (CR). This adaptive longevity effect was mediated by a specific hepatic proteomic signature that underscores the energy–splicing resilience axis hypothesis of aging (Ferrucci et al. 2022), which proposes that dietary strategies beyond CR and fasting (both considered benchmark interventions for lifespan extension) may achieve analogous beneficial outcomes (Figure 1). Since sustained adherence to CR is often difficult to achieve, the translational implications of these findings are significant. The study of Brandon et al. proposes an alternative non‐mitochondrial intervention to extend lifespan, with benefits obtainable by direct modulation of the spliceosomal machinery. We discuss how alternative splicing (AS) emerges in aging both as a target of age‐related error accumulation and a mediator of adaptive resilience.

**FIGURE 1:**
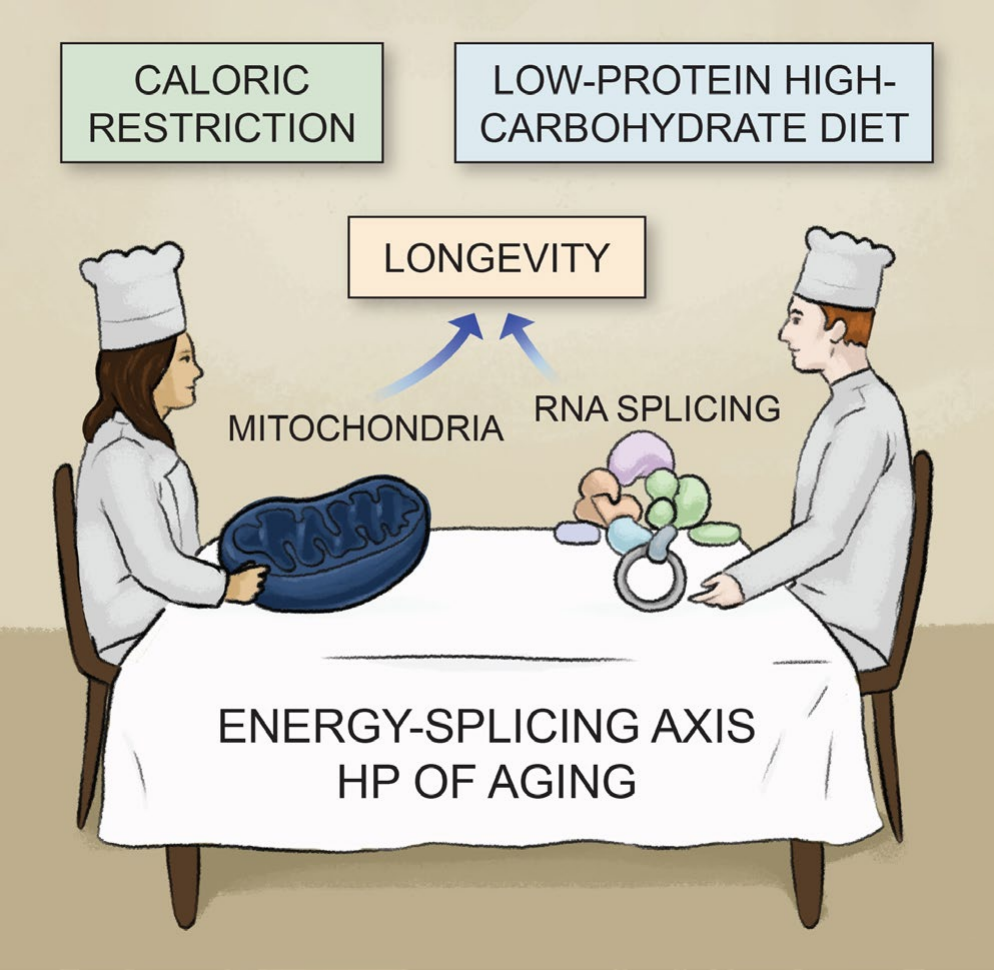
Distinct dietary interventions converge on longevity through the energy–splicing axis. Both caloric restriction (CR), primarily enhancing mitochondrial function, and a low‐protein, high‐carbohydrate (LPHC) diet, modulating RNA splicing homeostasis, promote longevity. These parallel yet complementary mechanisms support the “energy–splicing axis” hypothesis of aging.

## 1 LPHC Diet Triggers Adaptive Splicing to Improve Energy and Nutrient Status

In their work, Brandon et al. show that the hepatic proteome of mice fed a low‐protein, high‐carbohydrate (LPHC) diet was enriched in spliceosome components and nuclear pre‐mRNA processing factors, in contrast to CR, which mainly increased mitochondrial‐related proteins. They further observed altered isoform expression of SRSF1 (serine/arginine‐rich splicing factor 1, crucial in gene expression influencing mRNA stability), suggesting that AS responds directly and dynamically to energy and nutrient status without requiring direct mitochondrial intermediation. The latter strengthens the notion that splicing is an adaptable, resilience‐promoting mechanism, rather than only a passive fatality of aging. Other studies previously demonstrated that dietary regimes characterized by low protein‐to‐carbohydrate ratios affect lifespan and AS independently of caloric intake, with significant tissue‐specific effects (Wahl et al. 2018; Tabrez et al. 2017; Kim et al. 2025). These findings align with the “Geometric Framework for Nutrition” model that proposes that total energy intake is driven by protein leverage (Simpson et al. 2017). However, an important translational caveat must be noted: although protein restriction can extend lifespan in model organisms, in humans it frequently drives an overall increase in calorie consumption to meet protein targets, potentially weakening the intervention unless satiety pathways can be effectively modulated. Mechanistically, these findings support the energy‐splicing resilience axis hypothesis of aging, which integrates nutrient sensing, energy availability, and AS‐mediated homeostasis: while mitochondrial function, AMPK signaling, and splicing factor regulation converge to maintain adaptive capacity during aging, they are not obligatorily interconnected in homeostasis (Ferrucci et al. 2022). Splicing emerges then as an energetically demanding, yet crucial mechanism for resilience and tissue maintenance in the face of rising molecular disorder. In turn, functional modulation of splicing factors and RNA‐binding proteins through different pathways can preserve physiological function and extend health span (Gao and Jia 2025; Tao et al. 2024; Baralle and Romano 2023).

CR increases mitochondrial and associated proteins involved in energy metabolism, whereas LPHC diets increase proteins regulating AS and pre‐mRNA processing, particularly SRSF1. In line with this view, AMPK‐mediated phosphorylation of SRSF1 directly links energy availability to RNA binding and splicing activity, demonstrating a molecular conduit through which diet and pharmacological agents such as metformin can influence AS (Matsumoto et al. 2020). Metformin's AMPK activation has been shown to modulate SRSF1 function and mitigate pathological splicing in models of premature aging. Animal studies further support a causal role for AS in longevity regulation. In *C. elegans* , dietary restriction induces intron retention in ribosomal mRNAs, slowing translation and promoting survival, while suppression of age‐related intron retention in metabolic genes correlates with enhanced lifespan. Similarly, in *C. elegans*, the homolog for human SF1, which is the splicing factor SFA‐1, modulates dietary restriction–mediated longevity via TORC1‐dependent mechanisms (Heintz et al. 2017).

We have previously shown that, in human skeletal muscle, the age‐related decline in mitochondrial mass (as assessed by measuring mitochondrial protein abundance and oxidative capacity) is accompanied by an increase in spliceosome proteins (Ubaida‐Mohien et al. 2019; Zampino et al. 2020). In contrast, physical activity in octogenarian master athletes is associated with widespread downregulation of spliceosome components (Ubaida‐Mohien et al. 2022). At the transcriptomic level, mRNAs encoding splicing factors are enriched only in the low‐fitness cohort, as determined in human muscle biopsies from cross‐sectional studies (Donega et al. 2024). Given this evidence, we propose that conditions of energy scarcity may activate compensatory biological mechanisms mediated by the transcription and translation of alternative proteoforms. Because CR modulates energy metabolism and mitochondrial function, it is not surprising that, consistent with the energy–splicing resilience axis hypothesis, other authors have reported increased AS activity in response to CR (Green et al. 2022). These findings highlight the tissue‐ and context‐specific nature of AS in the adaptive response to energy availability.

## 2 Resilience‐Promoting AS and Potential Challenges

While its role in aging research remains underestimated and poorly understood, AS contributes to cellular resilience by generating proteomic diversity through selective exon inclusion or exclusion, maintaining cellular homeostasis and ensuring survival following stress, energetic perturbations, and metabolic shifts (Deschenes and Chabot 2017). At the same time, the spliceosome machinery is susceptible to age‐related damage accumulation leading to cellular dysfunction through errors causing intron retention, exon skipping, and cryptic exon activation (Bhadra et al. 2020; Rodriguez et al. 2016). Comparative studies across species show that changes in the levels and types of splicing factors expressed correlate with lifespan in both mice and humans (Lee et al. 2016). These changes create an AS paradox in aging, namely that in late life, the period when resilience is most crucial, splicing fidelity declines most sharply. Indeed, regulating splicing is only one dimension in the larger spectrum of changes to ensure cell maintenance; these changes reflect a complex interplay between adaptive compensation and dysregulated error (Angarola and Anczukow 2021; Ule and Blencowe 2019). Moreover, the spliceosome itself becomes increasingly vulnerable to damage, and therefore inefficiency, blurring the distinction between adaptive and maladaptive RNA splicing.

Transcriptomic studies have found a gradual decline in the accuracy of mRNA splicing reactions linked to cancer, immune dysfunction, and neurodegeneration (Ren et al. 2021). In particular, changes in mRNA splicing in the aging brain show increased intron retention, mainly affecting pre‐mRNAs encoding metabolic and DNA repair proteins (Adusumalli et al. 2019). Studies in human brain further show that aging and neurodegeneration are associated with widespread splicing alterations (Tollervey et al. 2011). The finding that aberrant splicing creates neoantigens offers a mechanistic bridge between splicing dysregulation and immune aging, as these neoantigens may accelerate immunosenescence and neuroinflammation (Li et al. 2025). Aberrant splicing of disease‐associated proteins directly implicated in neuronal pathology, such as UNC13A in TDP‐43, exemplifies maladaptive AS events, while increased intron retention in metabolic proteins may represent evolved adaptive responses (Brown et al. 2022).

The effect of LPHC diets on longevity reported by Brandon et al. illustrates nicely this aging splicing paradox, that is, a health‐promoting diet may provide a practical ad libitum approach to modulating the energy‐splicing resilience axis. By targeting both mitochondrial metabolism and splicing, such strategies may enable personalized interventions that optimize AS patterns to preserve healthy proteomes in older populations.

To be able to design therapies directed at the spliceosome with the goal of slowing or improving aging, it will be essential to answer three key questions. First, how can adaptive, resilience‐promoting splicing be confidently distinguished from maladaptive events? Second, how does AS change over time in different tissues? Third, how do specific dietary interventions, macronutrient ratios, or pharmacologic modulators influence splicing outcomes? We propose that the answers will emerge from multi‐omics approaches, functional validation in multiple model systems, and careful consideration of tissue‐specific effects (Yu et al. 2022). Ultimately, intervention studies are essential for distinguishing adaptive from maladaptive splicing, optimizing dietary and pharmacologic modulation, and establishing AS as a therapeutic target for promoting healthy longevity. With its positive and detrimental impacts, AS exemplifies both the vulnerability and the adaptive potential in aging: AS decline contributes to molecular disorder, while modulating AS may be leveraged to increase resilience.

## Author Contributions

S.D. and L.F. drafted the manuscript, S.D. drafted the figure, and L.F., S.D., and M.G. finalized the manuscript.

## Funding

This research was supported by the Intramural Research Program of the National Institutes of Health (NIH).

## Conflicts of Interest

The authors declare no conflicts of interest.

## Data Availability

Data sharing not applicable to this article as no datasets were generated or analyzed during the current study.
